# Efficacy of immunotherapy in *HER2*-mutated non-small cell lung cancer: a single-arm meta-analysis

**DOI:** 10.1007/s00432-023-05509-0

**Published:** 2024-01-27

**Authors:** Juguang Zhang, Weizhong Han, Jun Guo, Chufeng Zhang, Lijun Cao, Lixiu Peng, Xiao Han, Zhehai Wang

**Affiliations:** 1 Department of Medical Oncology, Shandong Cancer Hospital and Institute, Shandong First Medical University and Shandong Academy of Medical Sciences, Jinan, Shandong China; 2 Department of Cardiology, Shandong Provincial Hospital Affiliated to Shandong First Medical University, Jinan, Shandong China; 3 Department of Oncology, Liaocheng Chiping District Hospital of Traditional Chinese Medicine, Liaocheng, Shandong China; 4 Department of Internal Medicine, Qilu Hospital of Shandong University, Jinan, Shandong China

**Keywords:** Non-small cell lung cancers, *HER2*, Immune checkpoint inhibitors, Efficacy, Meta-analysis

## Abstract

**Background:**

Non-small cell lung cancers (NSCLC) harboring *Human Epidermal Growth Factor Receptor 2 (HER2*) mutations represent a distinct subset with unique therapeutic challenges. Although immune checkpoint inhibitors (ICIs) have been transformative in lung cancer treatment, the efficacy of ICIs in *HER2*-mutated NSCLC remains to be established.

**Methods:**

We systematically searched for real-world studies investigating the use of ICIs in treating *HER2*-mutated NSCLC, sourced from the PubMed, Cochrane Library, and Embase databases. Outcomes including objective response rate (ORR), disease control rate (DCR), and progression-free survival (PFS) were extracted for further analysis.

**Results:**

Twelve studies involving 260 patients were enrolled in this meta-analysis. Pooled data revealed an ORR of 0.26 (95% CI 0.17–0.34), a DCR of 0.68 (95% CI 0.55–0.81), and a median PFS (mPFS) of 5.36 months (95% CI 3.50–7.21). Notably, in the subgroup receiving combined immune and chemotherapy, the ORR increased to 0.37 (95% CI 0.26–0.49), the DCR to 0.79 (95% CI 0.70–0.87), and the mPFS to 7.10 months (95% CI 5.21–8.99).

**Conclusions:**

ICIs demonstrate promising anti-tumor activity and safety in patients with *HER2*-mutated NSCLC. Furthermore, the combined regimen of ICIs and chemotherapy may provide a significant therapeutic option for this patient population.

## Introduction

Lung cancer remains a significant global health burden as one of the principal causes of cancer-related mortality, with its prevalence steadily increasing in recent years. Non-small cell lung cancer (NSCLC) represents approximately 80%–85% of all lung cancer cases (Planchard et al. 2018; Sung et al. 2021). *The Human Epidermal Growth Factor Receptor 2 (HER2),* a tyrosine kinase receptor of the *ERBB/HER* family, has emerged as a critical regulator of cell growth and differentiation. Despite lacking a specific endogenous ligand, it activates the downstream PI3K-AKT and MEK-ERK signaling pathways to promote cell proliferation(Cheng et al. 2016; Ricciardi et al. 2014; Yu et al. 2022). Abnormalities in *HER2* signaling can result from *HER2* mutations, amplification, or protein overexpression, with *HER2* mutations identified in 1%–4% of NSCLC cases(Pillai et al. 2017).

Chemotherapy has been the cornerstone treatment for *HER2*-mutated NSCLC, with previous studies highlighting a median progression-free survival (mPFS) of 4.3 months for chemotherapy alone, 6.2 months for pemetrexed + platinum/bevacizumab, 2.6 months for gemcitabine, 4 months for paclitaxel + platinum/bevacizumab, and 3.5 months for vincristine. However, the mPFS for *HER2* tyrosine kinase inhibitors (TKIs) stands at a mere 2.2 months (Eng et al. 2016; Wang et al. 2018). Despite concerted efforts in recent years, clinical studies focusing on *HER2*-targeted therapy for *HER2*-positive NSCLC have yielded unsatisfactory results, with such NSCLC receiving only limited clinical benefit from targeted therapy(Uy, Merkhofer, & Baik, 2022). Therefore, alternative treatment strategies are desperately needed for patients with *HER2*-mutated NSCLC. Notably, immune checkpoint inhibitors (ICIs)-based therapy has demonstrated substantial advances in NSCLC treatment in recent years. Both ICIs alone or in conjunction with chemotherapy currently considered conventional treatments for NSCLC, showing some benefits over chemotherapy (Nasser et al. 2020). Nonetheless, the effectiveness of immunotherapy in NSCLC patients with *HER2* mutations remains unclear. The low prevalence of the mutation and the minor patient sample sizes have hindered the execution of extensive randomized controlled clinical trials. As such, the present study aims to fill this knowledge gap by conducting a meta-analysis of published real-world studies to evaluate the efficacy and safety of ICIs in treating patients with *HER2*-mutated NSCLC.

## Materials and methods

### Search strategy

Three databases (PubMed, Embase, and the Cochrane Library) were comprehensively searched for relevant studies. The date of the last search was 31 October 2022. Our searches incorporated subject terms like "non small cell lung cancer," "*HER2*, *ErbB-2*," "Immune Checkpoint Inhibitors," alongside free terms like "NSCLC," "ICI," "ICIs," "ICPI," "Rare targetable drivers (RTDs)" and terms with similar meanings.

### Selection criteria

The inclusion criteria for studies in this analysis were as follows: (1) involved real-world data on the efficacy or safety of ICIs for *HER2*-mutated NSCLC, (2) included participants who were 18 years or older with histologically or cytologically confirmed NSCLC, and *HER2* mutations as confirmed by tumor tissue testing or liquid biopsy, (3) presented sufficient data or data that could be calculated for efficacy and/or safety outcome indicators. We excluded studies that were reviews, conference abstracts, or other non-peer-reviewed literature, repetitive studies where the same clinical data was published more than once (we used the most recent, comprehensive version), publications in languages other than English, studies that did not provide enough data for extraction, and studies of low quality.

### Data extraction

Two reviewers independently screened studies, extracted data, and cross-verified the data. Extracted data included: (1) basic information like the first author, year of publication, and country of origin, (2) clinical baseline data such as the study population, sample size, PDL-1 expression status, treatment regimen, the number of lines of ICIs used, smoking history, gender, age, and (3) outcome indicators including objective response rate (ORR), disease control rate (DCR), median progression-free survival (mPFS), median overall survival (mOS), and safety outcome indicators such as the incidence of adverse events and incidence of grade 3–5 adverse events.

### Quality assessment

Two reviewers evaluated the quality of the studies using the MINORS scale. This scale, designed for the assessment of non-randomized studies, involves eight indicators each rated on a scale of 0–2, for a total possible score of 16. High-quality studies were defined as those scoring 13–16 points, medium-quality studies as those scoring 9–12 points (these were considered for final inclusion and data extraction), and low-quality studies as those scoring less than 9 points (these were excluded).

### Statistical analysis

We used Stata 17.0 software for our data analysis. We calculated the ORR and DCR using the combined ratio method, with ES as an effect size measure and a 95% confidence interval (CI). We estimated mPFS using the combined mean method, with the corresponding 95% CI. Heterogeneity among the studies was evaluated, with I^2^ ≤ 25% indicating low heterogeneity and 25% < I^2^ ≤ 50% indicating moderate heterogeneity; both were analyzed using a fixed-effects model. Studies with I^2^ > 50% (indicating high heterogeneity) were analyzed using a random-effects model. For results with high heterogeneity, we conducted a subgroup analysis. In cases of insufficient statistics or excessive heterogeneity, we performed descriptive analyses. The data were combined and presented as forest plots. To examine publication bias in the meta-analysis, we used visual funnel plots and quantitatively using Egger's test, with p < 0.05 considered statistically significant. In cases where publication bias was suggested, we further evaluated the effect using the 'cut-and-fill' method. We assessed the stability of the results through sensitivity analysis.

## Results

### Literature selection and basic characteristics of included studies

**Fig. 1:**
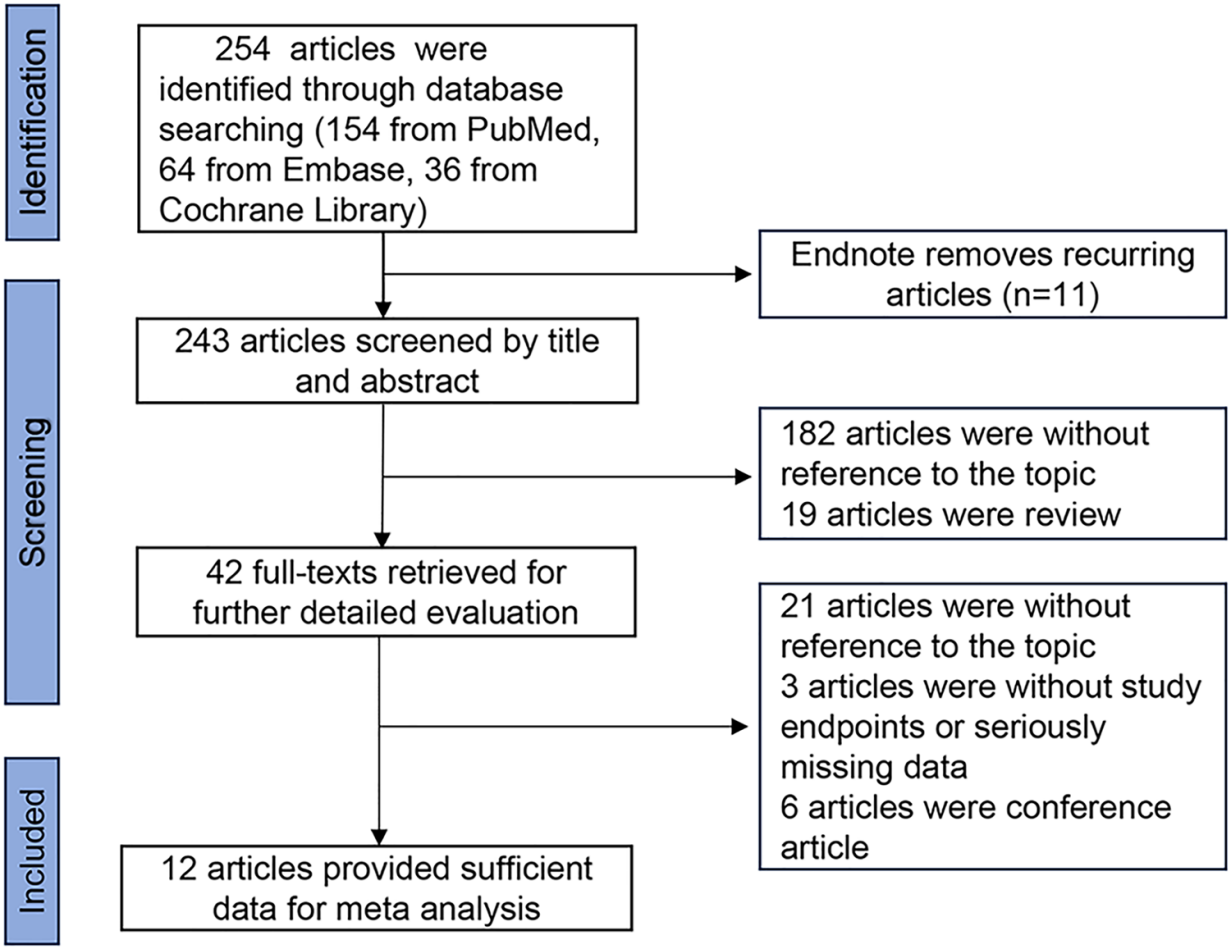
Literature search results and specific screening process

An initial search yielded 254 articles imported into Endnote for further management. Duplicates were removed using Endnote, followed by careful examination of the titles, abstracts, and full texts. Twelve real-world retrospective cohort studies were ultimately chosen for inclusion in our meta-analysis after this process resulted in the exclusion of studies that did not meet the inclusion criteria. The flow of study selection is presented in Fig. 1.

### Characteristics of included studies

Twelve included studies offered at least one validity index and were real-world investigations that complied with the established inclusion and exclusion criteria. These studies included 260 patients, most of whom were in stages III or IV, reflecting a patient population with advanced NSCLC. The principal characteristics of the included studies are provided in Table 1.

**Table 1:** Information on the articles eligible and their specific characteristics

Author	Region	Total number of patients	Median age	Smoking history	Staging	Type of Her-2 mutation	PD-L1 status	Treatment plan	ICIs	ORR	DCR	mPFS (months)	mOS (months)	Safety	MINORS score
Elizabeth Dudnika (2018)	Israel	7 (13)	NA	NA	NA	ERBB2	NA	ICIs	≥ 2nd	14%	NA	3.4(95% CI 2.4–8.5)	17.5 (95% CI:3.0–17.5)	NA	13
J. Mazieres (2019)	Europe	29	62	44.8%	IV	Exon 20	53.3% > 1%	93%ICIs	Most ≥ 2nd	7.4%	33.3%	2.5 (95% CI 1.8–3.5)	20.3 (7.8–NR)	NA	14
Florian Guisier (2020)	France	23	62.8	35%	IV	Exon 20 ins	17.4% > 1%	ICIs	≥ 2nd	27.3%	50%	2.2(95% CI 1.7–15.2)	20.4 (95% CI 9.3-NA)	AEs 26% AEs ≥ Grade 3 10%	15
Panwen Tian (2021)	China	13	55	53.8%	IV	Exon 20ins	NA	ICIs + Chemo	1st (*n* = 10)	31%	77%	8.0 (95% CI:5.2-NA)	NA	NA	13
									2nd (*n* = 3)						
Marcelo V Negrao (2021)	United States	21	NA	NA	NA	Codons 755 and 770–785	NA	ICIs	NA	NA	NA	3.02 (95% CI 1.8-NA)	10.81 (95% CI 5.62-NA)	NA	13
Xiangling Chu (2022)	China	26	55	30.8%	NA	Codons 755 and 770–785	26.9% > 1%	ICIs + Chemo	1st (*n* = 9) ≥ 2nd(*n* = 17)	38.5%	84.6%	7.4(95% CI 4.4–10.4)	NA	AEs 7(26.9%) AEs ≥ Grade 3 3(11.5%)	14
								ICIs							
								ICIs + antiangiogenic therapy							
Xiaojin Guo (2022)	China	12	59	33.3%	IV	Exon 20ins	16.7% > 1%	ICIs + Chemo	1st (*n* = 6) ≥ 2^nd^ (*n* = 6)	16.7%	91.7%	7.8(1–26.9)	NA	NA	13
								ICIs							
								ICIs + antiangiogenic therapy (1)							
Sally C.M. Lau (2021)	Canada	14	65	0	NA	Exon 20 ins and exons 17 and 19	57.1% > 1%	ICIs	1^st^ *n* = 3) ≥ 2nd (*n* = 11)	29%	57%	3.6(95% CI 1.6-NR)	NA	AEs 19%; AEs with high grade: 6%	16
Shuang Zhao (2021)	China	3	52	33.3%	IV	Exon 20	NA	ICIs + Chemo	1st	33.3%	66.7%	7 (2–8)	NA	NA	12
Felix C. Saalfeld (2021)	Germany	5	61	54.1%	III, IV	Exons 8, 19 and 20	50.8% > 1%	ICIs	1st	52%	76%	6 (95%CI:6–14)	1 year OS: 88%	NA	15
		22						ICIs + Chemo	1st						
		34						ICIs	≥ 2nd	16%	42%	4 (95%CI 4–6)	10(95% CI 6–NA)		
Guangjian Yang (2022)	China	46	55.4	36.9%	NA	Most Her-2 mutation	30.4% > 1%	ICIs + Chemo	1st	28.9%	80.%	5.20 (95% CI:3.64–6.76)	1 year OS: 53.3%	NA	15
Fawzi Abu Rous(F. 22)	United States	5	60	40%	IV	Exon 20,23	40% > 1%	ICIs + Chemo	NA	60%	80%	9	24	All AEs Grade 1/2	12

*PD-L1* Programmed Cell Death Ligand 1, *ORR* objective response rate, *DCR* disease control rate, *mPFS* median progression-free survival, *OS* overall survival, *mOS* median overall survival, *ins* insertions, *AEs* adverse reactions, *Chemo* Chemotherapy, *NA* not available, *NR* not reach. Note: The efficacy evaluation of all studies was the Response Evaluation Criteria in Solid Tumors (RECIST), and the adverse events evaluation of all studies was the Common Terminology Criteria for Adverse Events (CTCAE)

### Quality assessment of included studies

The MINORS scale was used to rate the 12 retrospective studies that were a part of our analysis. Ten studies were found to be of high quality, and two studies were of medium quality. Table 1 presents the quality assessment. The quality assessments are provided in Table 1.

### Meta-analysis results

#### Efficacy

**Fig. 2:**
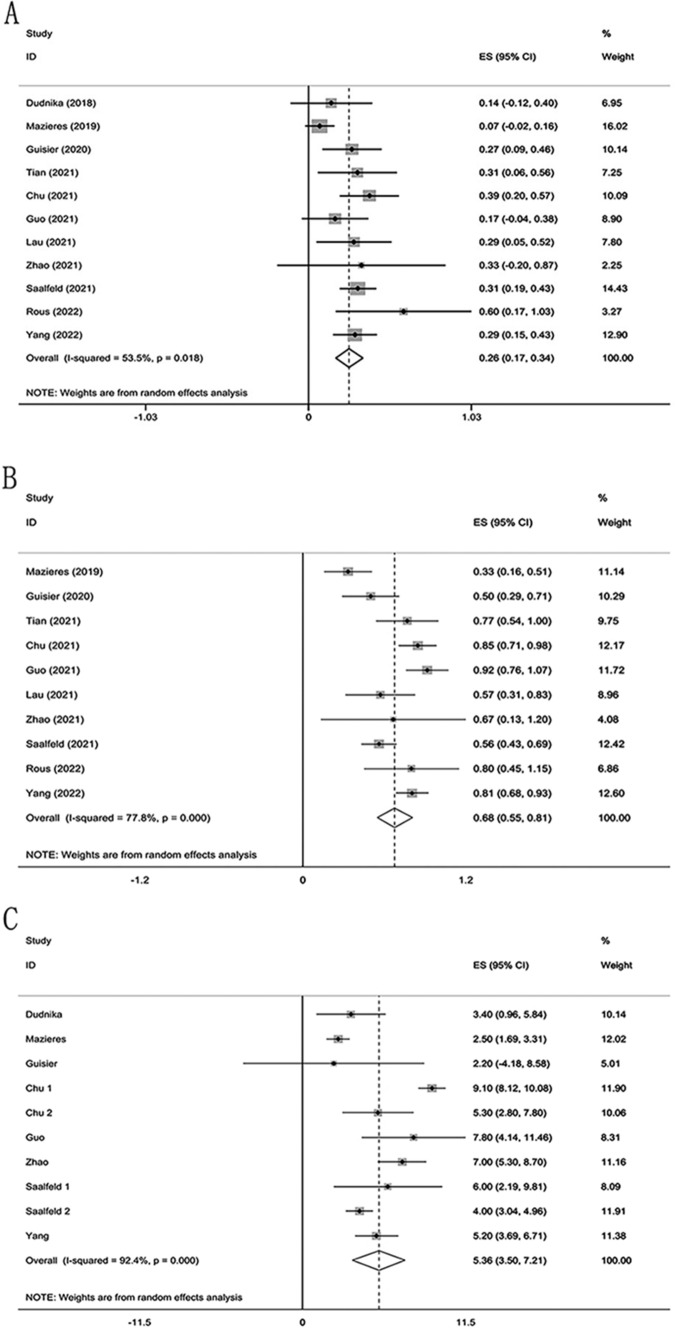
Pooled analysis of overall efficacy of *HER2*-mutated NSCLC: A ORR, B DCR, C mPFS

Eleven studies documented objective response rates (ORRs) (Abu Rous et al. 2022; Chu et al. 2022; Dudnik et al. 2018; Guisier et al. 2020; Guo et al. 2022; Lau et al. 2021; Mazieres et al. 2019; Saalfeld et al. 2021; Tian et al. 2021; Yang et al. 2022; Zhao et al. 2021). The meta-analysis revealed statistical heterogeneity across these studies (*P* = 0.018, *I*^2^ = 53.3%). Therefore, a random-effects model was utilized for the combined analysis. The pooled ORR was 0.26 (95% CI 0.17, 0.34) across 239 patients, as shown in Fig. 2.

Ten studies with DCR reported were used for the meta-analysis (Abu Rous et al. 2022; Chu et al. 2022; Guisier et al. 2020; Guo et al. 2022; Lau et al. 2021; Mazieres et al. 2019; Saalfeld et al. 2021; Tian et al. 2021; Yang et al. 2022; Zhao et al. 2021). The meta-analysis demonstrated significant heterogeneity across these studies (*P* = 0.000, *I*^2^ = 77.8%), leading us to employ a random-effects model for the combined analysis. The pooled DCR, as displayed in Fig. 2, was determined to be 0.68 (95% CI 0.55, 0.81) across 232 patients.

All of the twelve studies reported mPFS. However, many did not provide accurate confidence intervals. After careful deliberation, a meta-analysis of mPFS for the eight studies that presented complete data was carried out (Chu et al. 2022; Dudnik et al. 2018; Guisier et al. 2020; Guo et al. 2022; Mazieres et al. 2019; Saalfeld et al. 2021; Yang et al. 2022; Zhao et al. 2021). We used a random-effects model for the combined analysis because the meta-analysis revealed high heterogeneity among these studies (*P* = 0.000, *I*^2^ = 92.4%). The results, shown in Fig. 2, indicated a pooled mPFS of 5.36 months (95% CI 3.50, 7.21) for the 207 patients.

Although seven studies reported OS results, only one provided comprehensive OS data and precise confidence intervals, thus, performing a meta-analysis of OS was not feasible.

#### Safety

An acceptable safety profile was observed in four studies that reported adverse events. One study provided a detailed account of adverse events. Specifically, in a cohort of 26 patients with *HER2-*mutant NSCLC receiving ICIs, seven patients (26.9%) experienced adverse events. Among them, three patients (11.5%) had grade 3 to 4 adverse events, which included neutropenia, thrombocytopenia, and abnormal liver function, each occurring in one patient (Chu et al. 2022).

#### Subgroup analysis

Given the observed heterogeneity in the overall meta-analysis results, we further scrutinized four factors that could potentially contribute to this heterogeneity: treatment regimen, number of treatment lines, ethnic group, and age. Through this subgroup analysis, while the overall heterogeneity remained, we could derive more stable results with reduced heterogeneity within certain characteristic groups. Detailed findings are presented in Table 2.

**Table 2:** Subgroup analysis of the efficacy of ICIs in HER2-mutated NSCLC

	Grouping basis	Group	Number of articles	Number of patients	ES (95% CI)	I^2^/%	P	Total of I^2^/%	Total of *P*
ORR	Treatment options	ICIs	5	112	0.16 (0.07,0.24)	32.1	0.208	62.7	0.004
		ICIs + chemotherapy	5	83	0.37 (0.26,0.49)	11.4	0.341		
	Treatment lines	First line	5	93	0.36 (0.27,0.46)	34.5	0.177	58.9	0.007
		Second line or above	6	124	0.18 (0.09,0.26)	0	0.47		
	Ethnic group	Western	6	139	0.24 (0.11,0.37)	68	0.008	53.3	0.018
		Asian	5	95	0.29 (0.20,0.39)	0	0.675		
	Age	≥ 60 years	5	132	0.25 (0.11,0.40)	0	0.675	57.2	0.013
		< 60 years	5	95	0.29 (0.20,0.39)	74.2	0.004		
DCR	Treatment options	ICIs	4	105	0.44 (0.34,0.54)	0	0.442	73.2	< 0.001
		ICIs + chemotherapy	5	83	0.79 (0.70,0.87)	0	0.984		
	Treatment lines	First line	5	93	0.78 (0.69,0.86)	0	0.885	73.8	< 0.001
		Second line or above	5	117	0.51 (0.36,0.66)	64.1	0.025		
	Ethnic group	Western	5	132	0.52 (0.40,0.64)	44.8	0.123	77.8	< 0.001
		Asian	5	95	0.84 (0.76,0.91)	0	0.732		
	age	≥ 60 years	5	132	0.52 (0.40,0.64)	44.8	0.732	77.8	< 0.001
		< 60 years	5	95	0.84 (0.76,0.91)	0	0.123		
mPFS	Treatment options	ICIs	5	110	3.49 (2.42,4.56)	51.8	0.081	92.4	< 0.001
		ICIs + chemotherapy	5	92	7.10 (5.21,8.99)	79.8	0.001		
	Treatment lines	First line	4	80	6.97 (4.82,9.12)	84.8	0	93.1	< 0.001
		Second line or above	5	110	3.49 (2.42,4.56)	51.8	0.081		
	Ethnic group	Western	4	120	3.41 (2.33,4.49)	48	0.104	92.4	< 0.001
		Asian	4	82	6.91 (5.03,8.79)	82.2	0		
	Age	≥ 60 years	3	113	3.46 (2.13,4.79)	82.2	0	93.1	< 0.001
		< 60 years	4	82	6.91 (5.03,8.79)	60.8	0.054		

#### Publication bias

**Fig. 3:**
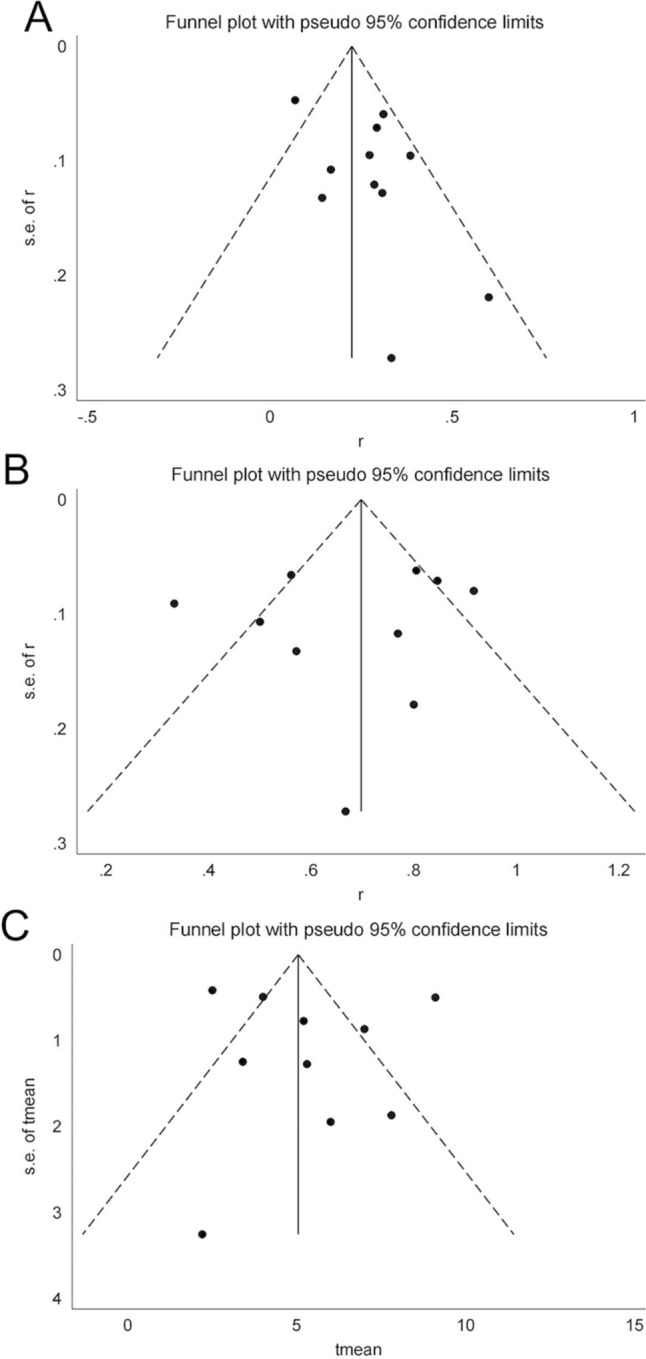
The funnel plot of ORR, DCR, mPFS: A ORR, B DCR, C mPFS

**Fig. 4:**
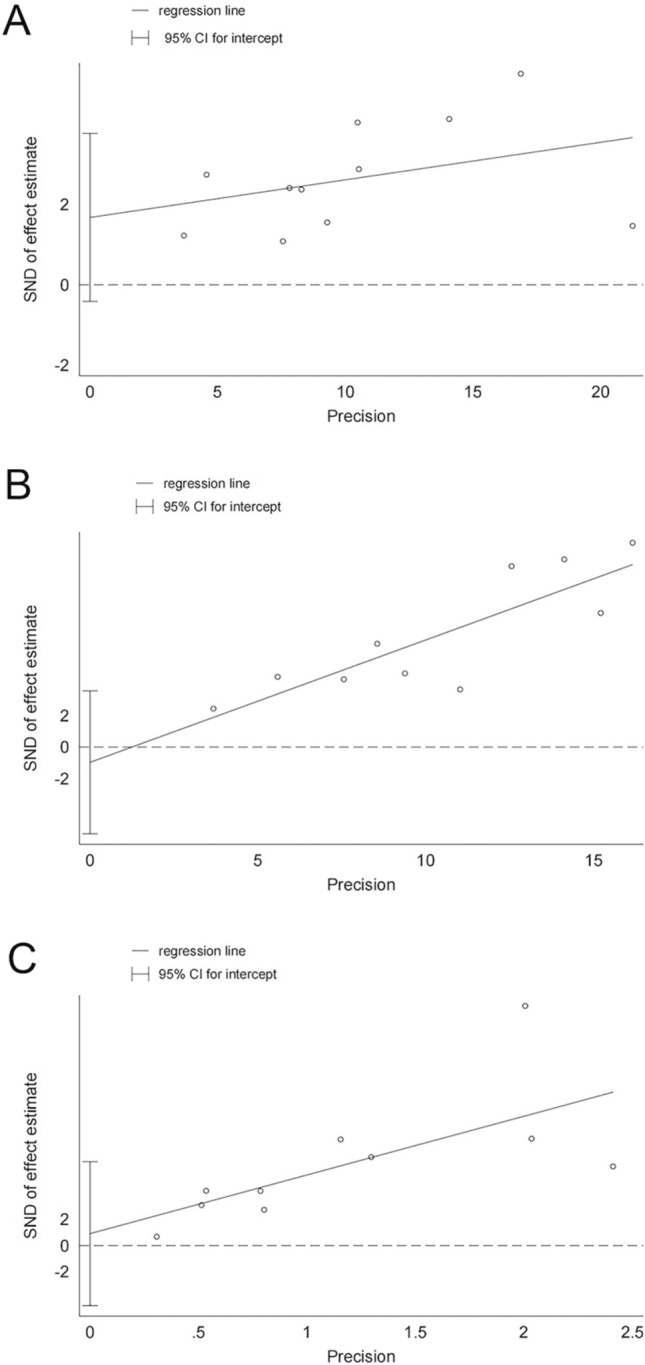
Egger linear regression plot of ORR, DCR, mPFS: A ORR, B DCR, C mPFS

The assessment of publication bias is presented in Fig. 3. A funnel plot illustrated that the majority of included studies fell within the inner side of the funnel, suggesting minimal publication bias among the selected literature. This observation was further quantified using Egger's test. The calculated p values for ORR, DCR, and mPFS were 0.103, 0.636, and 0.712, respectively, supporting that our results were not significantly influenced by publication bias. These results are illustrated in Fig. 4.

#### Sensitivity analysis

**Fig. 5:**
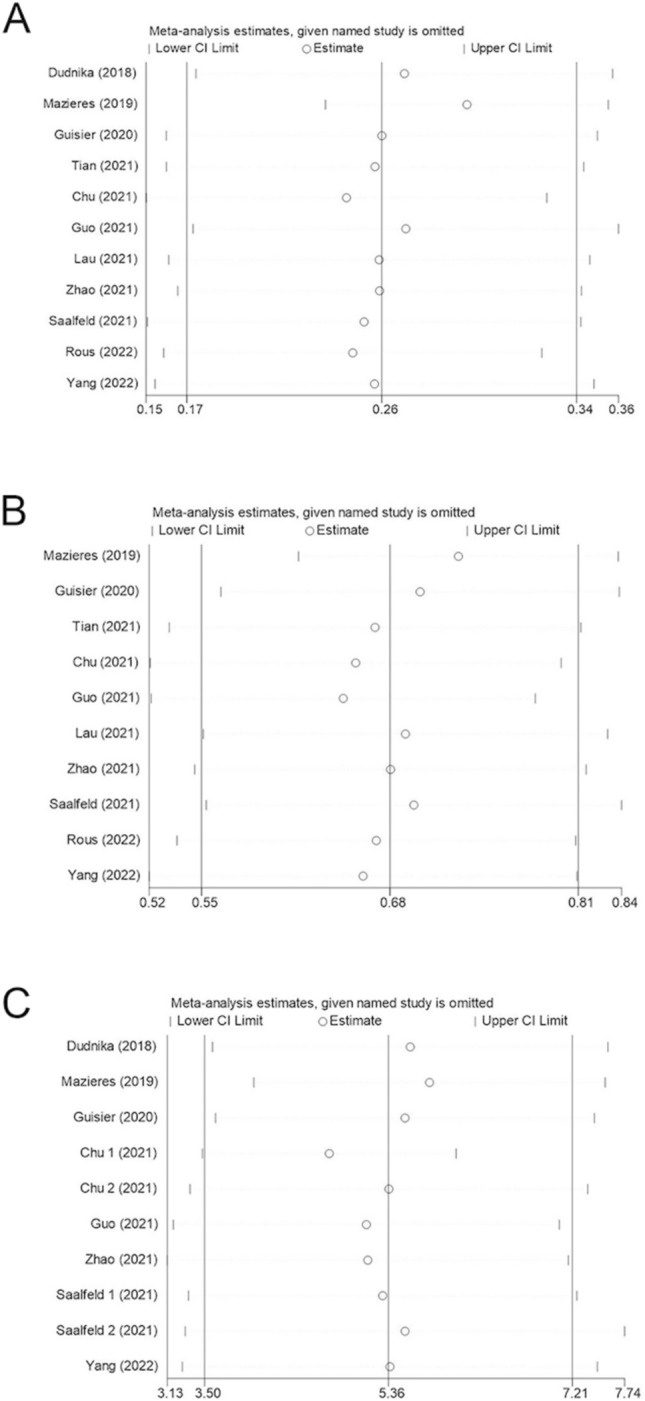
Sensitivity analysis of subsequent testing for impact on overall results: A ORR, B DCR, C mPFS

To assess the robustness of our meta-analysis results and to check if any individual study had a disproportionate influence on the overall results, we performed a sensitivity analysis by sequentially excluding one study at a time. Following this process, we noted only minor alterations to the overall results, suggesting that our findings are stable and reliable. These analyses are presented in Fig. 5.

## Discussion

HER2 mutations in NSCLC predominantly manifest in specific patient subsets, primarily women, never-smokers, and those diagnosed with adenocarcinoma women, never-smokers, and adenocarcinoma patients (Pillai et al. 2017). The primary therapy for advanced *HER2*-mutant NSCLC patients involves chemotherapy and targeted therapy, with chemotherapy remains the standard of care. Nevertheless, the efficacy of first-line chemotherapy remains poor, with an ORR of 36% and mPFS of 5.1 months (Wang et al. 2018). A retrospective analysis has disclosed a first-line chemotherapy objective response rate and progression-free survival rate of 43.5% and 6 months, respectively. These numbers dropped to 10% and 4.3 months for second-line chemotherapy and even less for third-line and beyond (Mazières et al. 2016).

Clinical interest in *HER2*-targeted therapies for patients with *HER2*-positive NSCLC has grown significantly in recent years. Clinical trials of *HER2*-targeted drugs like afatinib, neratinib, and dacomitinib yielded unsatisfactory outcomes with ORRs ranging from 0 to 19% and mPFS of 2.8–5.5 months (Dziadziuszko et al. 2019; Hyman et al. 2018; Kris et al. 2015; Lai et al. 2019; Peters et al. 2018). *HER2* monoclonal antibodies (e.g., trastuzumab and pertuzumab) have shown efficacy in advanced *HER2* mutant breast cancer and gastric cancer. However, their efficacy in NSCLC is limited. Even the addition of chemotherapy has failed to yield significant clinical benefits (Herbst et al. 2007; Kinoshita et al. 2018; Langer et al. 2004; Lara et al. 2004). Recently, Trastuzumab Deruxtecan (T-DXd) has demonstrated superior survival outcomes and received accelerated approval by the FDA for treating patients with NSCLC carrying *HER2 (ERBB2)* mutations on August 11, 2022. Nevertheless, the high toxicity of T-DXd impedes its broader application in clinical studies and practice (Li et al. 2022; Tsurutani et al. 2020; Y. Yu, Yang, Li, & Fan, 2023). The Phase II study DESTINY-Lung trial showed encouraging results, with T-DXd demonstrating an ORR of 55% and mPFS of 8.2 months in previously treated patients with *HER2-*mutated NSCLC. However, treatment-related adverse events were almost universal, with interstitial lung disease being particularly problematic, affecting 24 patients (26%) and causing two deaths (Li et al. 2022). Another notable ADC, Trastuzumab emtansine (T-DM1), while showing an ORR of 38.1%, had a limited mPFS of 2.8 months in a focused study of NSCLC patients with *HER2* exon 20 insertion mutations (Iwama et al. 2022). Similarly, in a phase II clinical trial, pyrotinib, an irreversible small molecule inhibitor of *EGFR/HER2/HER4* receptors, showed an ORR of 30% and an mPFS of 6.9 months in 60 patients with platinum-treated advanced NSCLC with *HER2* mutations. However, the majority of patients (98.3%) experienced treatment-related adverse events (TRAEs), with 28.3% of patients experiencing severe TRAEs, leading to 23.4% of patients discontinuing treatment (Zhou et al. 2020). The ZENITH20-2 trial treated 90 patients with *HER2-*mutated NSCLC with poziotinib, showing moderate efficacy (ORR of 27.8% and mPFS of 5.5 months). However, TRAEs were observed in 97.8% of patients, with severe TRAE occurring in 75 patients (84.4%), resulting in dose reduction for 76.7% and discontinuation for 13.3% of patients (Le et al. 2022).

The advent of ICIs has particularly revolutionized the NSCLC treatment landscape. However, the utility of ICIs in patients with *HER2*-mutated NSCLC remains under debate. The comprehensive meta-analysis was designed to assess the efficacy and safety of ICIs in treating *HER2*-mutated NSCLC, thereby aiming to furnish a scientific foundation for clinical treatment plans. In the study, twelve real-world studies meeting the predefined inclusion and exclusion criteria were selected, involving a cohort of 260 NSCLC patients who received ICIs as monotherapy or in combination with other treatments. These studies collectively reported an ORR of 0.26 (95% CI 0.17, 0.34) and a DCR of 0.68 (95% CI 0.55, 0.81) for patients receiving ICIs. In addition, analysis of the eight studies produced a combined mPFS of 5.36 months (95% CI 3.50, 7.21). A subgroup analysis further suggested that an ICIs–chemotherapy combination may yield superior anti-tumor efficacy, with an ORR of 0.37 (95% CI 0.26, 0.49), a DCR of 0.79 (95% CI 0.70, 0.87), an mPFS of 7.10 months (95% CI 5.21, 8.99). Adverse events as reported in four of the selected studies suggest a reasonably acceptable safety profile. Therefore, early use of ICIs in combination with chemotherapy may be a more effective treatment modality with a manageable safety profile for patients with advanced NSCLC with HER2 mutations.

Despite the meta-analysis's promising results on the efficacy of ICIs in advanced NSCLC with *HER2* mutations, the results need further exploration. Studies of ICIs combined with ADCs are an especially promising avenue, as preclinical data suggest that drugs like T-DXd may augment T-cell activity, upregulate PD-L1 expression, increase the number of tumor-infiltrating CD8 + T cells, and enhance the expression of PD-L1 and MHC class I molecules on the surface of tumor cells. As a result, combination therapy with T-DXd and anti-PD1 antibodies may be more effective than treatment alone (Iwata et al. 2018). Therefore, the combination of ICIs with HER2-targeted therapy shows promise. There are various issues to consider, including the mode of combination therapy of ICIs with ADCs or selective HER2 TKI and with chemotherapeutic agents, the dosage of drugs administered, the optimal sequence of treatment, and the unique management of toxicity. The results from the ongoing randomized controlled phase III study evaluating T-DXd in combination with pembrolizumab chemotherapy in first-line therapy (DESTINY-Lung 04) (Bob et al. 2022), and the exploratory phase I studies evaluating ADCs in combination with ICIs in previously treated patients (NCT04686305, NCT04042701, NCT05482568) may provide valuable insights for improving the treatment outlook for patients with *HER2*-mutated advanced NSCLC treated with ICIs in combination(Y. Yu et al. 2023).

This study has several limitations. First, there were insurmountable heterogeneity issues possibly arising from differences in patient age, gender, PD-L1 expression level, dosing regimen, drug type, and patient ethnicity between studies. Second, the limited amount of literature included in the subgroup analysis, coupled with the fact that most of the real-world studies retrieved were uncontrolled retrospective studies; Therefore, statistical methods could not be used to calculate relative hazard ratios between subgroups.

In conclusion, ICIs in combination with chemotherapy may represent a promising treatment modality for patients with *HER2*-mutated NSCLC, as this combination therapy has demonstrated promising efficacy and a manageable safety profile. The results of this study may serve as a reference for future clinical studies.

## Data Availability

The datasets used and analyzed during the current study are available from the corresponding author upon reasonable request.
